# Surgical Management of Left Sinus of Valsalva Aneurysm Presenting with Exertional Chest Pain

**DOI:** 10.21470/1678-9741-2019-0437

**Published:** 2020

**Authors:** Ali Gurbuz, Sahin Iscan, Orhan Gokalp, Erturk Karaagac, Senem Girgin, Murat Aksun

**Affiliations:** 1 Department of Cardiovascular Surgery, Izmir Katip Celebi University Ataturk Training and Research Hospital, Izmir, Turkey.; 2 Department of Anesthesiology, Izmir Katip Celebi University Ataturk Training and Research Hospital, Izmir, Turkey.

**Keywords:** Aortic Aneurysm, Aortic Valve Insufficiency, Myocardial Infarctation, Chest Pain, Sinus of Valsalva, Middle Aged, Male

## Abstract

Left sinus of Valsalva aneurysm (SVA) is a very infrequent clinical entity. Valsalva aneurysms are often asymptomatic in right and non-coronary sinuses and the diagnosis is often incidental. A left SVA which presents with exertional chest pain due to compression of left coronary system arteries is extremely rare. In this case, we present a successful surgical repair of left SVA without aortic regurgitation or myocardial infarction in a 59-year-old male patient.

**Table:** 

Abbreviations, acronyms & symbols	
AR	= Aortic regurgitation
CABG	= Coronary artery bypass grafting
Cx	= Circumflex artery
LAD	= Left anterior descending artery
LMCA	= Left main coronary artery
SVA	= Sinus of Valsalva aneurysm

## INTRODUCTION

Sinus of Valsalva aneurysms (SVAs) are rare anomalies and they are often asymptomatic. In most of the cases, the first symptom is related to aortic regurgitation (AR) or rupture. Rupture may cause fistulization to cardiac chambers or acute heart failure^[1]^. But it is uncommon a left SVA presenting with angina symptoms due to compression of left main coronary artery (LMCA), left anterior descending artery (LAD), and circumflex artery (Cx). With this case, we present a patient who had a giant left SVA and acute cardiac symptoms due to the elongation and the attenuation of the proximal left coronary system arteries.

## TECHNIQUE

**Fig. 1:**
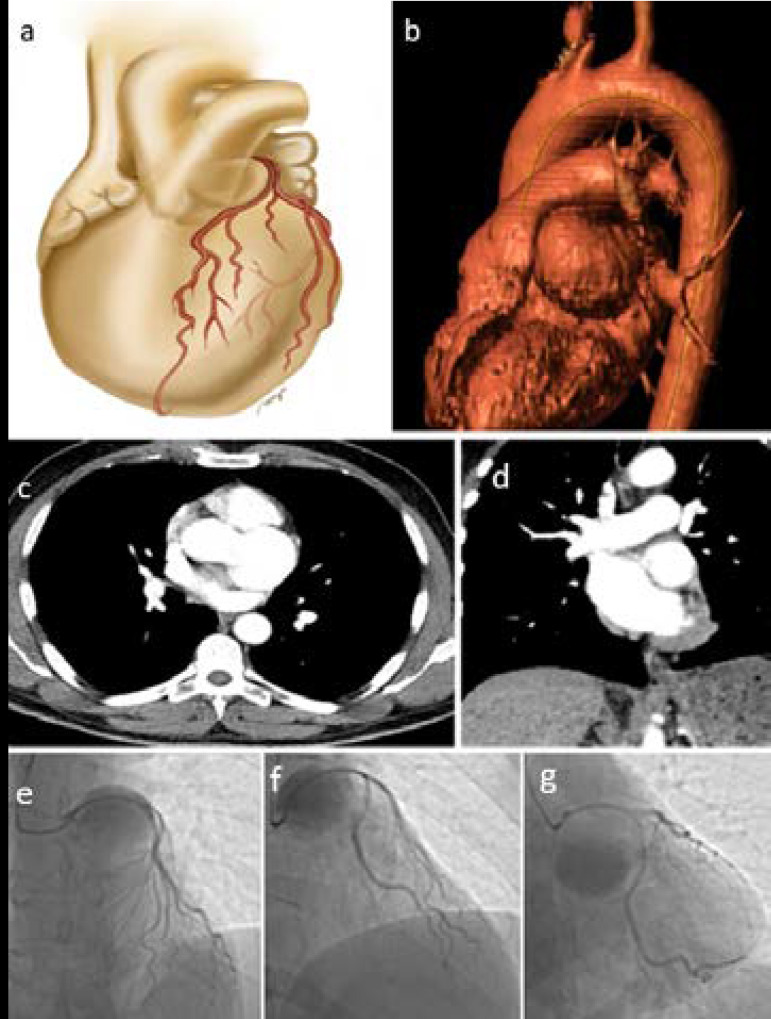
A and B) Illustration of sinus of Valsalva aneurysm (SVA), flattened left main coronary artery, and circumflex artery. Left SVA was located posteriorly to the pulmonar artery and it compressed coronary arteries by pushing them. C and D) Computed tomography images show saccular aneurysm (45 mm) originated from the left sinus of Valsalva. E to G) Cardiac catheterization shows narrowed left coronary system arteries.

**Fig. 2:**
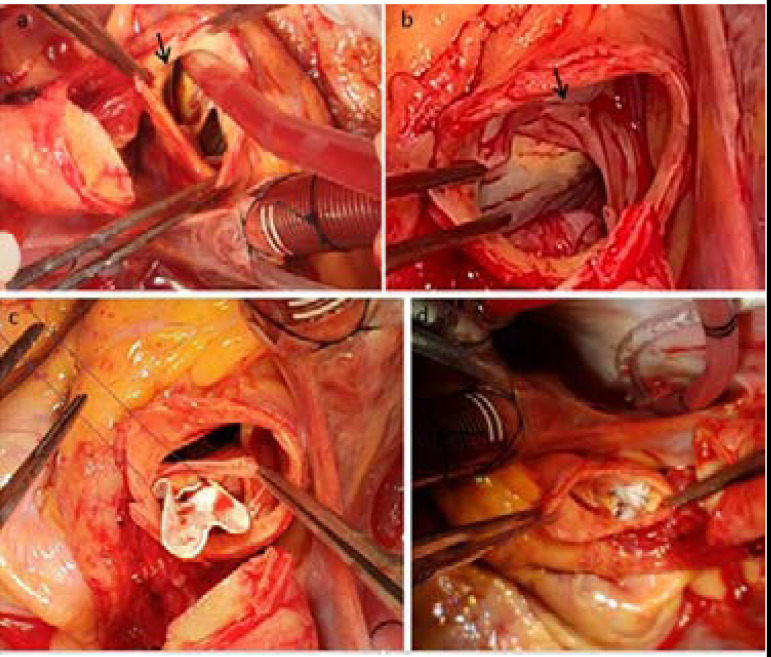
A and B) Sinus of Valsalva aneurysm with large orifice in the left coronary annulus. a) Left coronary button is very close to aneurysm sac with a thin rim (arrow). B) Arrow shows left coronary leaflet of aortic valve. C and D) Aneurysm was repaired with Gore-tex patch. Valvular site was sutured with U-stayed Prolene pledgeted sutures. Aortic site was sutured with continued technique.

A 59-year-old male was admitted to our hospital with a history of exertional chest pain for one month. The cardiac catheterization showed seriously compressed and elongated LMCA, LAD, and Cx by the mass effect of SVA. Echocardiography showed mild AR. Computed tomography scan showed a 45-mm SVA (Figure 1). The patient was operated on the next day. After the sternotomy, the left internal thoracic artery was harvested for grafting. Cardiopulmonary bypass was initiated with ascending aortic and right atrial cannulation. The aorta was completely transected on the supracoronary level. The inspection of the left sinus of Valsalva revealed a giant saccular aneurysm which contained a thin-proximal rim of LMCA (Figure 2). The intramural section of LMCA and Cx got thinner and they were flattened due to compression of the aneurysm. During the procedure, the origin of the left main trunk was ligated with Prolene sutures. Aortic leaflet coaptation was sufficient and there was no annular deformation. A Gore-Tex patch was sutured with U-stayed 5/0 Prolene pledgeted sutures, which were placed from the inferior to the superior part of the left coronary annulus of the aortic valve and the rest of the patch was sutured using continued 5/0 Prolene suture (Figure 2). The left internal thoracic artery was grafted to LAD and the saphenous vein grafts were used to bypass the first diagonal and the first obtuse marginal coronary arteries. The patient was weaned from the cardiopulmonary bypass with no AR. The patient was extubated six hours after the surgery. He was discharged home on the 7^th^ day. There was no valvular pathology on the postoperative echocardiography control and the first and fourth months of follow-up.

## DISCUSSION

SVAs are rare cardiac disorders in Western countries (0,1-3%), but its incidence has been reported five times greater in Asians^[2,3]^. Congenital defects may play a role in aortic tissue elasticity. Endocarditis, atherosclerosis, and trauma may also cause SVA less frequently^[4,5]^. SVA commonly originates from right and non-coronary sinus (99%). Its occurrence in the left sinus is very infrequent. In a study with Asian population, from 286 ruptured patients included, two cases (0,7%) were reported as left SVA, and another study in our country showed three left SVA cases in 53 patients without myocardial ischemia^[4,5]^. In this case, we present a left SVA which compress LMCA, LAD, and Cx. Our patient was admitted to the hospital with exertional chest pain, and cardiac catheterization showed non-ruptured SVA compressing the left coronary system arteries. All these complaints and findings, which constitute the major symptoms of acute coronary syndrome, are quite rare for SVA. The relation of SVA with LMCA is also interesting in this case.

When we searched through the literature, there were few cases diagnosed with both left SVA and myocardial ischemia due to compression of left coronary system arteries. In a case by Sarkar et al.^[6]^, a 64-year-old male patient with anteroseptal myocardial infarction due to LMCA and LAD compression and AR was presented. Bio-Bental procedure and LAD-Cx-coronary artery bypass grafting (CABG) were performed. In our case, there was no myocardial infarction or AR. The patient was admitted with myocardial ischemia symptoms and he was incidentally diagnosed with SVA. In another case, Pedroza et al.^[7]^ presented a 52-year-old female patient with myocardial infarction due to proximal LMCA compression and AR. Emergency valve-sparing root replacement and LAD-CABG were performed. Finally, Jiang et al.^[8]^ presented a patient with acute worsening chest pain due to middle LAD occlusion with the compression of the left SVA. They performed patch plasty for SVA and LAD-CABG. In our patient, the giant SVA compressed LMCA, LAD, and Cx with a mass effect. He underwent elective surgical repair of the left SVA with patch plasty technique. Our patient differentiates from other cases because of his intra-aneurismal location of the left main coronary trunk’s proximal section and the long segment compression of LMCA, LAD, and Cx. The position of this SVA is extremely rare. So, he was not suitable for LMCA reanastomosis and he needed a left coronary artery system bypass surgery. The origin of the left main trunk was sutured, and later internal thoracic artery-LAD; saphenous vein-first diagonal and saphenous vein-first obtuse marginal coronary bypasses were performed. Long-term continued external compression of vascular tissue also causes luminal narrowing and endothelial damage on the coronary arteries. Repair of SVA and coronary decompression was not sufficient for coronary remodeling. Concomitant revascularization is recommended in these patients^[8]^. There was also no AR in our patient. This advantage makes the procedure easier without valvular repair or aortic root repair. But, the need for the triple coronary bypass for all left coronary system is the complicated and interesting part of our surgery.

There are multiple surgical methods for the repair of SVA, including primary repair, patch closure, composite, and valve-sparing root replacement. Small aneurysms may be repaired in a primary fashion, but it does not seem sufficient for giant aneurysms. Patch closure rate for SVA repair is 50% to 90% in different studies^[5,8]^. Valve-sparing root replacements in experienced centers may be good surgical option for young patients with concomitant AR. In our patient with giant SVA, patch plasty was performed. There was no AR on the postoperative echocardiography control, and the first- and fourth-month follow-up echocardiographies of the patient were uneventful.

## CONCLUSION

To our knowledge, this is a very rare case of giant SVA which lead to left coronary artery system compression due to the space-occupying effect of the aneurysm mass and the partial involvement of the left main coronary trunk in the aneurysm wall while there was no AR. In these cases, it is important to keep in mind that full coronary revascularization may be necessary and, if possible, aortic valve preservation and valve preservation techniques can be applied.

**Table:** 

Authors' roles & responsibilities	
AG	Substantial contributions to the conception or design of the work; analysis or interpretation of data for the work; final approval of the version to be published
SI	Substantial contributions to the conception or design of the work; analysis of data for the work; final approval of the version to be published
OG	Substantial contributions to the conception or design of the work; analysis of data for the work; final approval of the version to be published
EK	Analysis of data for the work; final approval of the version to be published
SG	Analysis of data for the work; final approval of the version to be published
MA	Analysis of data for the work; final approval of the version to be published
